# Possible Roles of CC- and CXC-Chemokines in Regulating Bovine Endometrial Function during Early Pregnancy

**DOI:** 10.3390/ijms18040742

**Published:** 2017-03-31

**Authors:** Ryosuke Sakumoto, Ken-Go Hayashi, Shiori Fujii, Hiroko Kanahara, Misa Hosoe, Tadashi Furusawa, Keiichiro Kizaki

**Affiliations:** 1 Division of Animal Breeding and Reproduction Research, Institute of Livestock and Grassland Science, National Agriculture and Food Research Organization (NARO), Ibaraki 305-0901, Japan; hayaken@affrc.go.jp (K.-G.H.); s.love.sweets.03@gmail.com (S.F.); kanahara@affrc.go.jp (H.K.); 2 Division of Animal Sciences, Institute of Agrobiological Sciences, National Agriculture and Food Research Organization (NARO), Ibaraki 305-8602, Japan; hosoe@affrc.go.jp (M.H.); tfuru@affrc.go.jp (T.F.); 3 Laboratory of Veterinary Physiology, Iwate University, Iwate 020-8550, Japan; kizaki@iwate-u.ac.jp

**Keywords:** cow, endometrium, chemokines, tissue culture, pregnancy

## Abstract

The aim of the present study was to determine the possible roles of chemokines in regulating bovine endometrial function during early pregnancy. The expression of six chemokines, including CCL2, CCL8, CCL11, CCL14, CCL16, and CXCL10, was higher in the endometrium at 15 and 18 days of pregnancy than at the same days in non-pregnant animals. Immunohistochemical staining showed that chemokine receptors (CCR1, CCR2, CCR3, and CXCR3) were expressed in the epithelial cells and glandular epithelial cells of the bovine endometrium as well as in the fetal trophoblast obtained from a cow on day 18 of pregnancy. The addition of interferon-τ (IFNT) to an endometrial tissue culture system increased CCL8 and CXCL10 expression in the tissues, but did not affect CCL2, CCL11, and CCL16 expression. CCL14 expression by these tissues was inhibited by IFNT. CCL16, but not other chemokines, clearly stimulated interferon-stimulated gene 15 (ISG15) and myxovirus-resistance gene 1 (MX1) expression in these tissues. Cyclooxygenase 2 (COX2) expression decreased after stimulation with CCL8 and CCL14, and oxytocin receptor (OTR) expression was decreased by CCL2, CCL8, CCL14, and CXCL10. Collectively, the expression of chemokine genes is increased in the endometrium during early pregnancy. These genes may contribute to the regulation of endometrial function by inhibiting COX2 and OTR expression, subsequently decreasing prostaglandin production and preventing luteolysis in cows.

## 1. Introduction

The establishment of pregnancy is the result of successful communication between the conceptus and the maternal endometrium. When animals become pregnant, the corpus luteum (CL) remains functional and the dynamics of prostaglandin (PG) F2α secretion from uterus in early pregnancy changes from that in the comparable stages of the estrous cycle [1,2,3]. At the time of recognition of pregnancy, the bovine conceptus produces interferon-τ (IFNT) to prevent luteolysis, which is induced by a pulsatile release of PGF2α from the uterus [2,3]. IFNT plays various roles in ruminants, such as uterine receptivity to implantation and differentiation of the conceptus [2,3,4]. Extensive research during the last decade, including global transcriptome studies, demonstrated the enrichment of immune-related genes, including interferon-stimulated genes (ISGs), in the endometrium of a pregnant cow compared to non-pregnant or cyclic heifers [5,6,7,8,9,10,11]. However, the physiological roles of these pregnancy-dependently regulated genes in the mechanism of maternal recognition are not yet fully understood. Understanding the role of the fetal-maternal interaction during early pregnancy might help to determine a way to improve reproductive efficiency and alleviate deficiencies.

Therefore, in the present study, we evaluated global gene expression patterns in the endometrium of pregnant and non-pregnant cows using a custom-made, 15 K bovine oligo DNA microarray analysis. Since the expression of messenger RNA of six chemokines, including CCL2, CCL8, CCL11, CCL14, CCL16, and CXCL10, drastically increased in the endometrium during early pregnancy, the effects of IFNT and fetal trophoblast-derived total protein on the expression of chemokine genes were subsequently evaluated. Furthermore, the effects of these chemokines and pregnancy-related substances, including IFNT, tumor necrosis factor-α (TNF) and leukemia inhibitory factor (LIF), on the expression of interferon-stimulated gene 15 (ISG15), myxovirus-resistance gene 1 (MX1), cyclooxygenase 2 (COX2), oxytocin receptor (OTR), and estrogen receptor α (ESR1) by cultured endometrial tissues were studied to assess the physiological roles of chemokines in regulating endometrial function during early pregnancy in cows.

## 2. Results

### 2.1. Expression of Chemokines and Their Receptors in the Bovine Endometrium during Early Pregnancy

Microarray analysis detected 344 and 1336 differentially expressed genes in the bovine endometrium at 15 and 18 days of pregnancy compared with the same days in non-pregnant cows (>2-fold change; *p* < 0.05). The expression of six chemokines, including CCL2, CCL8, CCL11, CCL14, CCL16, and CXCL10, was higher in the endometrium during early pregnancy than in the non-pregnant stage (Table 1).

**Figure 1:**
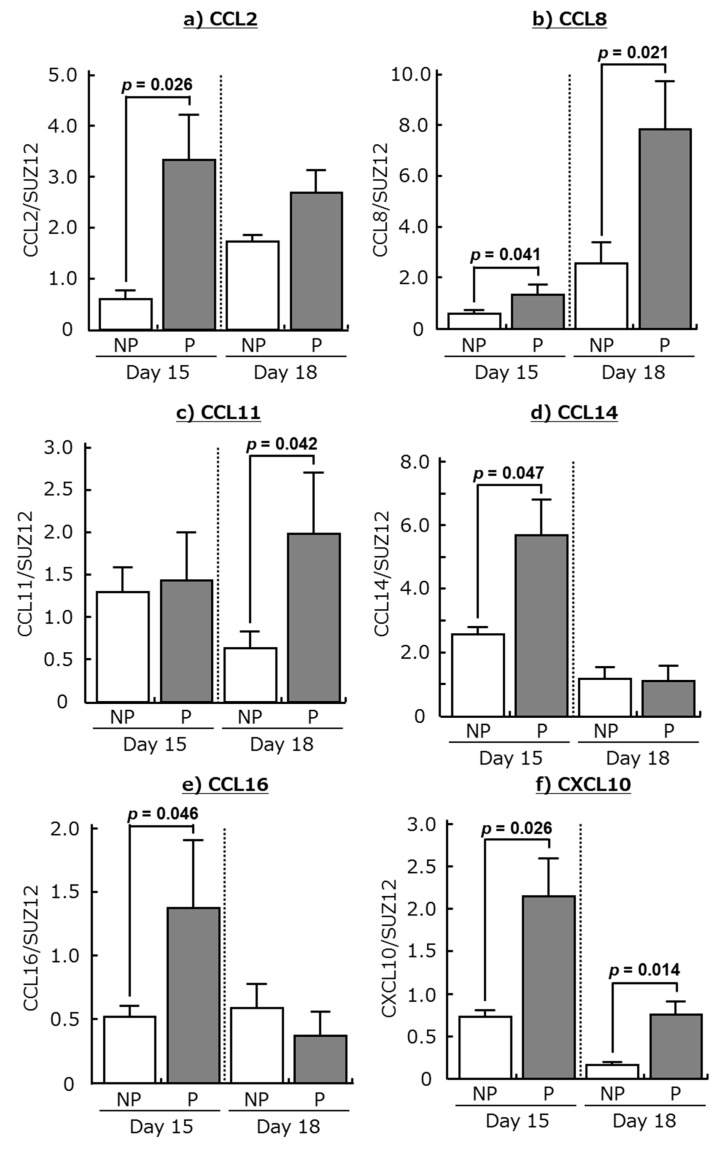
Changes in relative amounts of mRNA for (**a**) CCL2, (**b**) CCL8, (**c**) CCL11, (**d**) CCL14, (**e**) CCL16, and (**f**) CXCL10 in the endometrium at days 15 and 18 of non-pregnant cows (NP) and pregnant cows (P). Data are means ± SEM of four cows per stage and are expressed as relative ratios of the mRNAs to SUZ12 polycomb repressive complex 2 subunit (SUZ12). *p*-Values show significant differences between NP and P.

Messenger RNA expression of these chemokines was confirmed by real-time PCR. Transcripts of CCL2, CCL8, CCL14, CCL16, and CXCL10 were more abundant in the endometrium at day 15 of pregnancy than at day 15 in non-pregnant cows (Figure 1; *p* < 0.05). Moreover, the expression of CCL8, CCL11, and CXCL10 mRNA was higher in the endometrium at day 18 of pregnancy than at day 18 in non-pregnant cows (Figure 1; *p* < 0.05). Although messenger RNAs of chemokine receptors (CCR1, CCR2, CCR3, and CXCR3) were detected in the bovine endometrium during both the estrous cycle and pregnancy, there were no significant changes in the expression of these receptors between the estrous cycle and pregnancy.

**Figure 2:**
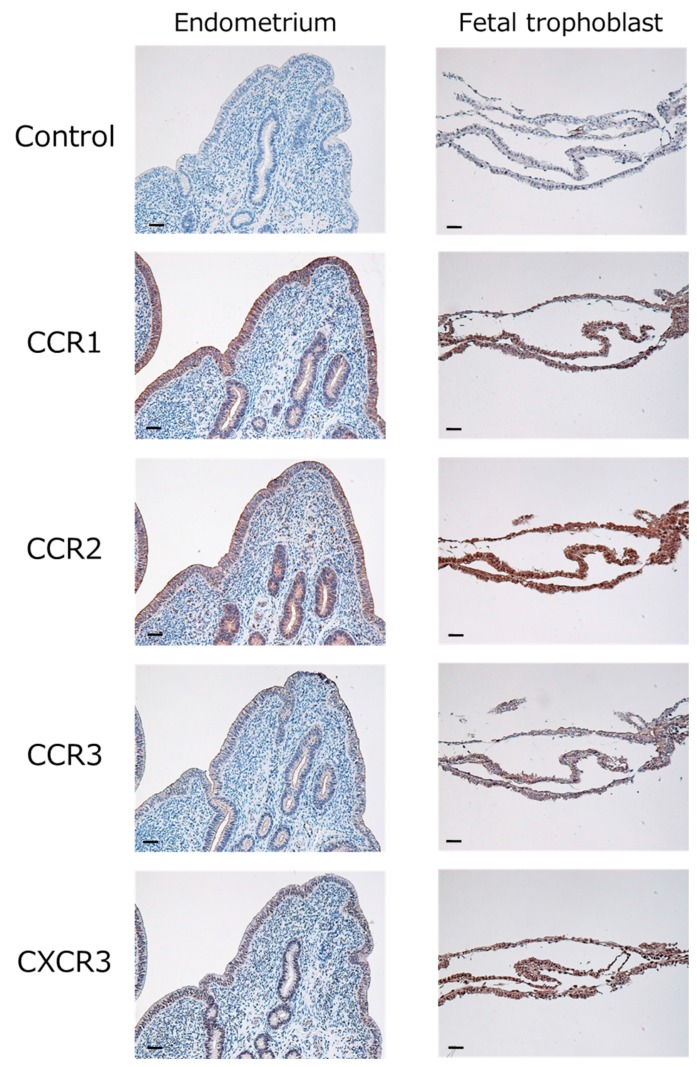
Localization of CCR1 (binds to CCL8, CCL14, and CCL16), CCR2 (binds to CCL2, CCL8, and CCL16), CCR3 (binds to CCL11), and CXCR3 (binds to CXCL10) in the bovine endometrium and fetal trophoblast obtained from cows in their 18th day of pregnancy. Intensive immunoreactivity was observed in endometrial epithelial cells, glandular epithelial cells, or fetal trophoblast. No positive immunoreactivity was observed in the negative control (Control). Scale bar = 50 µm.

Immunohistochemical staining showed that CCR1 (binds to CCL8, CCL14, and CCL16), CCR2 (binds to CCL2, CCL8, and CCL16), CCR3 (binds to CCL11), and CXCR3 (binds to CXCL10) were expressed in the epithelial and glandular epithelial cells of the endometrium as well as in the fetal trophoblast obtained from day 18 of pregnancy (Figure 2).

### 2.2. Effects of IFNT and FMP on Chemokine Expression in Cultured Endometrial Tissues

**Figure 3:**
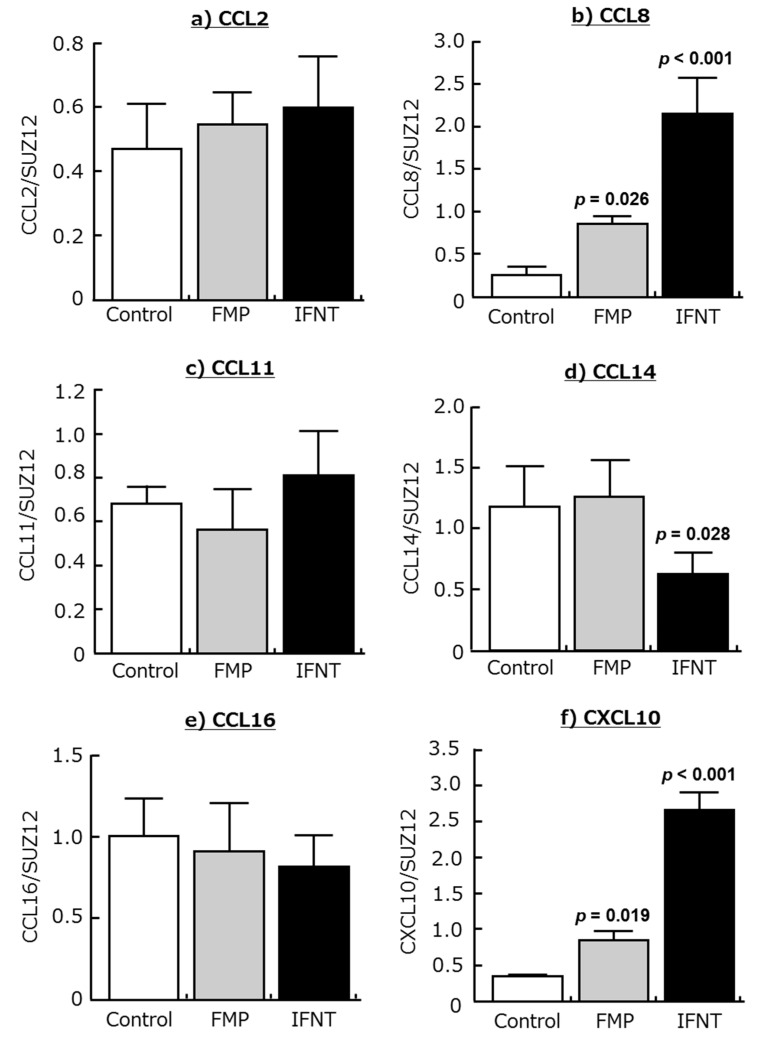
Effects of the supernatant derived from homogenized fetal trophoblast (FMP; 200 ng/mL) and interferon-τ (IFNT; 100 ng/mL) on the mRNA expression of (**a**) CCL2, (**b**) CCL8, (**c**) CCL11, (**d**) CCL14, (**e**) CCL16, and (**f**) CXCL10 in cultured bovine endometrial tissues. Homogenization buffer was added at the control group. Data are means ± SEM of five cows and are expressed as relative ratios of the mRNAs to SUZ12. *p*-Values show significant differences between treated group and control group.

The addition of IFNT increased CCL8 and CXCL10 expression in cultured endometrial tissue (Figure 3b,f; *p* < 0.01), but it did not affect CCL2, CCL11, and CCL16 expression. CCL14 expression in this tissue was inhibited by IFNT (Figure 3d; *p* < 0.05). In addition, the supernatant derived from homogenized fetal trophoblast (FMP) of day 18 of pregnancy stimulated CCL8 and CXCL10 expression in the endometrial tissues (Figure 3b,f; *p* < 0.05).

### 2.3. Effects of Chemokines, IFNT, FMP, TNF, and LIF on ISG15, MX1, COX2, OTR, and ESR1 Expression in Cultured Endometrial Tissues

**Figure 4:**
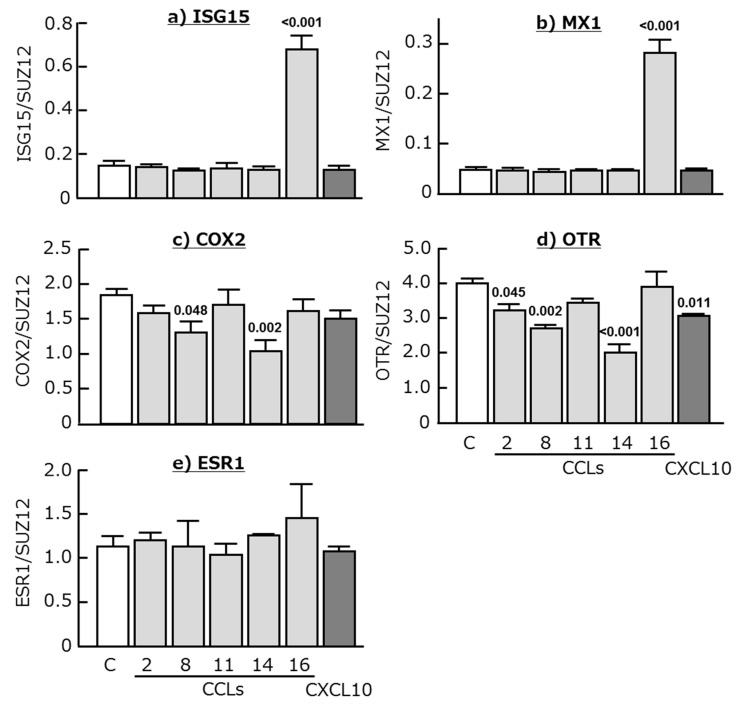
Effects of CCL2, CCL8, CCL11, CCL14, CCL16, and CXCL10 (50 ng/mL each) on the mRNA expression of (**a**) interferon-stimulated gene 15 (ISG15), (**b**) myxovirus-resistance gene 1 (MX1), (**c**) cyclooxygenase 2 (COX2), (**d**) oxytocin receptor (OTR), and (**e**) estrogen receptor α (ESR1) in cultured bovine endometrial tissues. Data are means ± SEM of five cows and are expressed as relative ratios of the mRNAs to SUZ12. *p*-Values show significant differences between treated group and control group.

CCL16 clearly stimulated both ISG15 and MX1 expression in the cultured endometrial tissues (*p* < 0.001), whereas the other chemokines had no effect (Figure 4a,b). COX2 expression decreased after the stimulation of CCL8 and CCL14 (Figure 4c; *p* < 0.05 or lower), and OTR expression was decreased by CCL2, CCL8, CCL14, and CXCL10 (Figure 4d; *p* < 0.05 or lower). In contrast, ESR1 mRNA expression was not affected by all tested chemokines (Figure 4e).

**Figure 5:**
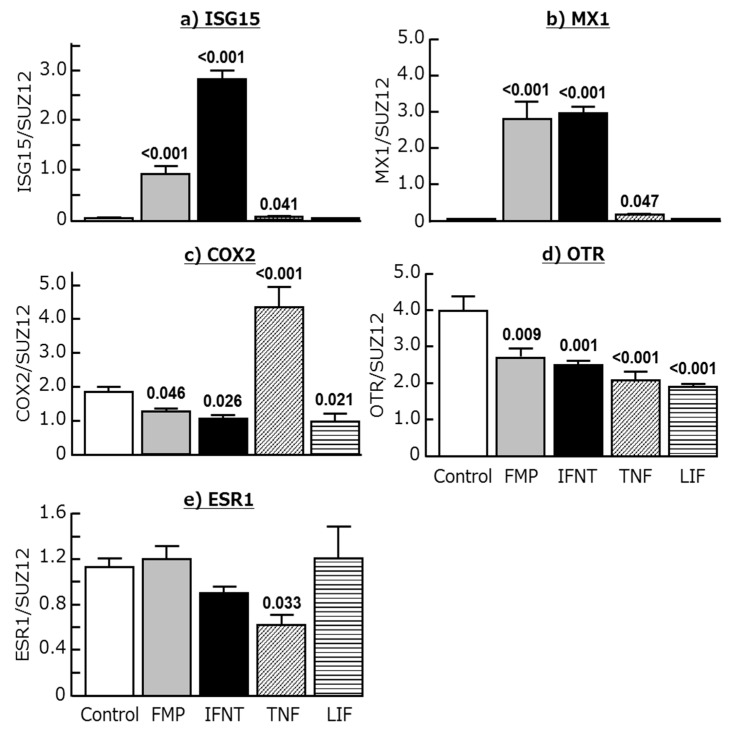
Effects of the supernatant derived from homogenized fetal trophoblast (FMP; 200 ng/mL), interferon-τ (IFNT; 100 ng/mL), tumor necrosis factor α (TNF; 50 ng/mL), and leukemia inhibitory factor (LIF; 50 ng/mL) on the mRNA expression of (**a**) ISG15, (**b**) MX1, (**c**) COX2, (**d**) OTR, and (**e**) ESR1 in cultured bovine endometrial tissues. Data are means ± SEM of five cows and are expressed as relative ratios of the mRNAs to SUZ12. *p*-Values show significant differences between treated group and control group.

The addition of IFNT and FMP increased the expression of ISG15 and MX1, and decreased the expression of COX2 and OTR, in cultured endometrial tissues (Figure 5a–d; *p* < 0.05 or lower). As expected, TNF stimulated COX2 expression and decreased OTR and ESR1 expression in these tissues (Figure 5c–e; *p* < 0.05). Interestingly, it also stimulated the expression of both ISG15 and MX1 (Figure 5a,b; *p* < 0.05). Moreover, LIF decreased the expression of COX2 and OTR in endometrial tissues (Figure 5c,d; *p* < 0.05), but did not affect ISG15, MX1, and ESR1 expression.

## 3. Discussion

The results of the current study demonstrated that gene expression in the bovine endometrium during early pregnancy differs from that in the endometrium during the estrous cycle. In particular, gene expression of six chemokines was apparently high in the endometrium on days 15 and 18 in pregnant cows in comparison to the non-pregnant cows. In this study, we focused on the endometrial gene expressions on days 15 and 18 of pregnancy since IFNT production from trophoblast drastically increases around days 14–15 [12], and elongated conceptus is implanted to endometrial epithelial cells immediately after (on days 18–19) in cattle [5]. Messenger RNA expression of CCL8 and CXCL10 was higher in the endometrium during both day 15 and 18 of pregnancy, but the expression of CCL2, CCL14, and CCL16 were high in the endometrium of day 15 of pregnancy. CCL11 expression in the endometrium was high in the endometrium during only day 18 of pregnancy, suggesting that stage dependent changes of the expression of these chemokines have multiple roles in regulating maternal-conceptus communication. Moreover, CCL8 and CXCL10 were increased by stimulation with IFNT and FMP using an in vitro culture system. The binding sites of chemokines were present in the endometrial epithelial and glandular epithelial cells. All tested chemokines, except for CCL11, attenuated the expression of genes coding for pregnancy-related substances in the cultured endometrial tissues. These observations lead us to hypothesize that chemokines play an important role in regulating bovine endometrial function during early pregnancy.

Immune cells, the main sources and targets of chemokines, are present in bovine endometrium throughout the estrous cycle, and the number of immune cells increases around the late luteal stage [13]. Eosinophils secrete both CCL2 (known as MCP-1) and CCL8 (MCP-2) and the number of eosinophils increase within the uterus during early pregnancy in ewes [14]. Both CCL2 and CCL8 act through CCR2 found on monocytes and memory T cells [15]. In addition, CCL8 also binds to CCR1 found primarily on T cells, monocytes, and eosinophils [15]. Here, we demonstrated that both CCR1 and CCR2 are present in endometrial epithelial cells, and that the expression of CCL8 was increased by IFNT and FMP, suggesting that CCL2 and CCL8 could affect endometrial function during the early gestation period. Indeed, the present study demonstrated that CCL2 decreases COX2 expression and CCL8 decreases both the expression of COX2 and OTR in cultured endometrial tissues. Since oxytocin (OT) stimulates endometrial PG production by activating COX2 in endometrial cells [16], CCL2 and CCL8 may subsequently contribute to inhibit PGF2α production to establish pregnancy in cows.

CXCL10, also known as IP-10, is found in monocytes that are localized in the subepithelial stroma of ovine endometrium, and its expression is stimulated by IFNT and interferon-γ [17]. The expression level of CXCL10 in the endometrium is higher in pregnancy than in the non-pregnant stage in goats [18]. In cows, CXCL10 expression in the endometrium was high in pregnancy and its mRNA expression was stimulated by both FMP and IFNT in the present study. Furthermore, CXCR3, which is the binding site of CXCL10, was expressed in the bovine endometrium and fetal trophoblast on day 18 of pregnancy. CXCL10 inhibits OTR expression in cultured endometrial tissues. A previous study demonstrated that CXCL10 could induce the recruitment of numerous leukocytes, lymphocytes, and/or monocytes to the ovine uterus and enhance the ability to attach to day 17 trophoblasts and day 20 chorionic membranes [19]. CXCL10 also induces caprine trophoblast adhesion [20] and chemotaxis in human trophoblast cell lines [21]. These findings suggest that CXCL10 might affect not only PG production from the endometrium, but also conceptus elongation at an early stage of pregnancy in cows.

In the present study, since CCL14 (also known as HCC-1) expression increased in the endometrium of pregnant cows, we expected that CCL14 expression of the cultured endometrial tissues would have been increased by stimulation with FMP or IFNT. However, FMP had no effect and IFNT adversely inhibited CCL14 expression. We could not find an appropriate explanation for this apparently contradictory phenomenon. Since chemokine production is regulated by many factors, including cytokines, growth factors, and steroids [17,22,23], other factor(s) may have powerful effects on the stimulation of CCL14 production in the endometrium of early pregnant cows. For example, although IFNT inhibited CCL14 expression in these tissues, FMP showed no effect in this study. Hence, some substances other than IFNT in FMP might indirectly stimulate CCL14 production in cultured endometrial tissues, causing the inhibitory effects of IFNT on CCL14 expression to be masked. In humans, CCL14 is produced maximally by the endometrium at the time of embryo implantation and during early pregnancy, predominantly by decidualized stroma and epithelial cells [24], and its receptor (CCR1) is present on invasive extravillous trophoblasts [25]. In addition, the migration of trophoblasts is stimulated by CCL14 [25]. Since CCR1 protein was expressed in the fetal trophoblast as well as in endometrial epithelial cells in this study, CCL14 may induce the promotion of trophoblast migration in cows. In this study, CCL14 reduced COX2 and OTR expression in cultured endometrial tissues, suggesting that CCL14 decreases PG production in the bovine uterus. Taken together, although further studies regarding the physiological impact of CCL14 in regulating endometrial function are required, CCL14 may be an important factor in maternal-fetal communication in cows.

Little information is available about the involvement of CCL11 in bovine reproduction. CCL11, also known as eotaxin-1, increased in the endometrium on day 18 of pregnancy in this study. Since the binding sites of CCL11 (CCR3) are expressed in bovine and human [26] endometrial epithelial cells, we expected that this chemokine would act as a paracrine factor in the endometrium after day 18 of pregnancy. However, CCL11 expression was not affected by FMP and IFNT, and CCL11 did not affect the expression of any gene in cultured endometrial tissues. A previous study demonstrated that CCL11 induces angiogenic responses by human CCR3-positive endothelial cells [27], and that estradiol-17β (E2) acts through CCL11 to recruit eosinophils to the uterine stroma during the estrous cycle in mice, but that these cells do not have a function in regulating either the duration of the estrous cycle or the fertility of mice [28]. In addition, CCL11 regulates extravillous trophoblast migration, invasion, and adhesion, highlighting a potential regulatory role for these chemokines during uterine decidual spiral arteriole remodeling in the first trimester of human pregnancy [29]. Thus, CCL11 may act on the trophoblast-conceptus via CCR3 rather than on endometrial cells in pregnant cows.

The precise roles of CCL16 in the bovine endometrium are currently unknown, but there is considerable information regarding its functions in immunological disease and cancer in humans. CCL16 mRNA was elevated in women with endometriosis, and immunoreactive CCL16 was predominantly demonstrated in the endometrium [30]. CCL16 increases antigen presentation of macrophages, enhances T-cell cytotoxicity, and stimulates the production of a number of inflammatory-type cytokines (interleukin-1β, TNF, interleukin-12) [31]. CCL16 and its receptors were identified in preterm placenta [32]. In addition, CCL16 induces endothelial cell motility, which is pivotal in vessel formation by stimulating the release of proinflammatory and proangiogenic chemokines [33,34]. In this study, CCL16 receptors (CCR1, CCR2) were expressed in the endometrial epithelial cells, and CCL16 drastically stimulated the expression of ISG15 and MX1 in cultured endometrial tissues. These effects are comparable with those of FMP and IFNT. Although a further study is needed to clarify the role of CCL16 in regulating bovine endometrial function in detail, it may positively influence angiogenesis and anti-viral activity by up-regulating ISG15 and MX1 expression at the time of maternal recognition in cows.

IFNT, the pregnancy recognition hormone in ruminants, is produced by mononuclear trophectoderm cells of conceptuses at a critical time to prevent regression of the CL function. One mechanism by which IFNT inhibits luteolysis is the down-regulation of OTR, which prevents OT-stimulated PGF secretion [35]. As expected, IFNT clearly stimulated ISG15 and MX1 expression but inhibited COX2 and OTR expression by the cultured endometrial tissues, suggesting that in vitro culture systems can support previous data regarding the biological effects of IFNT. On the other hand, TNF is well recognized as a potent luteolytic factor in many mammalian species, including cows. TNF and TNF receptors are present in the bovine endometrium, and TNF stimulates PGF production only in the bovine endometrial stromal cells by increasing COX2 mRNA in cattle [36,37,38]. IFNT reduced TNF-induced PGF synthesis directly by attenuating COX2 gene expression in bovine endometrial stromal cells in a dose-dependent manner [38]. Indeed, TNF stimulated COX2 and inhibited the expression of OTR and ESR1 in endometrial tissues in the present study. In contrast, TNF significantly stimulated the expression of both ISG15 and MX1 in endometrial tissues, similar to INFT, which is a known anti-luteolytic agent. Since TNF acts through TNF receptor type I/type II via various intracellular signaling pathways, including nuclear factor-kappa B/activator protein-1 and mitogen-activated protein kinase [39], TNF may contribute directly or indirectly to anti-viral activity within the uterus. LIF mRNA expression is observed in the endometrium of both humans and mice and its concentration increases in mid- and late secretory phases [40]. LIF is regarded as an important factor in both murine and human embryo implantation or decidualization. LIF-deficient female mice are infertile due to the failure of implantation [41]. Since LIF reduced COX2 and OTR expression in cultured endometrial tissues in this study, LIF may play an important role in establishing pregnancy in the process of maternal recognition in cows as well as in humans and rodents.

## 4. Materials and Methods

### 4.1. Collection of the Bovine Uterus and Fetal Trophoblast

Bovine uteri were obtained from Japanese Black cows at the institute ranch within 10–30 min of exsanguination. For the microarray analysis and immunohistochemistry, tissue samples were collected from cows on days 15 and 18 after artificial insemination (*n* = 4 animals/stage). The day of artificial insemination was designated as day 1. The uterine horn with ipsilateral of the CL was obtained and immediately cut open to see the endometrium. The presence or absence of fetal trophoblast was checked macroscopically to determine whether the cows were pregnant or not. The caruncular endometrial tissues (<0.5 cm^3^) were collected and submerged in RNAlater (Qiagen GmbH, Hilden, Germany) or in 10% neutral formalin and stored until use.

Supernatants derived from homogenized fetal trophoblast on day 18 of pregnancy (FMP) were collected as described previously [42]. Briefly, the fetal trophoblast was transferred into 2.5 mL of ice-cold homogenized buffer (300 mM sucrose, 25 mM Tris-HCl, 2 mM EDTA; pH 7.4) containing a proteinase inhibitor tablet (cOmplete Ultra tablet EDTA-free, Roche Diagnostics, Tokyo, Japan), and was homogenized in an ice bath with a rotor-stator homogenizer (TissueRuptor; Qiagen) using three 30 s bursts at maximum speed with 20 s intervals of cooling between each burst. The homogenate was subsequently centrifuged at 23,500× *g* for 30 min at 4 °C. The supernatant was collected and total protein concentration was measured using a commercial protein assay kit (DC Protein Assay Kit, 500-0111JA, BIO-RAD Laboratories Co., Ltd., Tokyo, Japan).

All procedures for animal experiments were carried out in accordance with guidelines approved by the Animal Ethics Committee of the National Institute of Agrobiological Sciences on 1 April 2014 (#H18-036-3).

### 4.2. Microarray Analysis

A custom-made 15 K bovine oligo DNA microarray was used for the microarray analysis, which was performed according to previous reports [43,44]. After verifying the quality of the RNA with a NanoDrop ND-1000 spectrophotometer (NanoDrop Technology Inc., Wilmington, DE, USA), and an Experion RNA StdSens kit (700-7104JA, BIO-RAD Laboratories), we performed one-color microarray analysis. RNA integrity was confirmed, and all samples had an A260/280 ratio greater than 1.8 and an RNA integrity number greater than 8.5. The oligomicroarray produced by Agilent Technologies (Palo Alto, CA, USA) was used in this study. Sixty-mer nucleotide probes for the customized microarray were synthesized on a glass slide. cDNA synthesis, Cy3-labeled cRNA preparation, hybridization, and the washing and scanning of the array slides were performed according to the Agilent one color microarray-based gene expression analysis protocol. Briefly, 400 ng of total RNA from each sample were reverse-transcribed into cDNA using the Quick Amp Labeling Kit (Agilent Technologies) with an oligo dT-based primer, and then Cy3-labelled cRNA was prepared by in vitro transcription. Labeled cRNA was purified with an RNeasy Mini Kit, and the concentration and Cy3 dye incorporation (pmol Cy3/µg cRNA) were measured with a spectrophotometer. Labeled cRNA (600 ng) was fragmented and hybridized using the Gene Expression Hybridization Kit (Agilent Technologies), according to the manufacturer’s instructions. The arrays were washed using a Gene Expression Wash Pack Kit and scanned using an Agilent Microarray Scanner. Feature Extraction ver. 9.5 was used for image analysis and data extraction. The microarray data from each sample were imported into GeneSpring 12 (Agilent Technologies) for use in the software’s normalization algorithm and for candidate gene detection. Normalization was performed by dividing each measurement of each array by the median of all measurements in that array (per chip normalization). The Gene Expression Omnibus (GEO, available online: http://www.ncbi.nlm.nih.gov/geo/query/acc.cgi) accession numbers are as follows: GPL9284 for the platform, GSM2455260 to GSM2455274 for the samples, and GSE93580 for the series.

### 4.3. Real-Time PCR

Total RNA isolation and subsequent reverse transcription and real-time PCR steps were carried out as previously described [45]. The primers encoding the bovine sequences were chosen using an online software package (available online: http://primer3.ut.ee/) and synthesized as listed in Table 2. The primer length (18–25 bp) and GC contents of each primer (50–60%) were selected to avoid primer dimer formation.

Gene expression was measured by real-time PCR using an Mx3000P Real time PCR analyzing system (Agilent Technologies) and a QuantiFast SYBR Green PCR kit (204054, Qiagen) starting with 500 ng of reverse-transcribed total RNA. PCR was performed under the following conditions: (first step) 95 °C for 5 min; 45 cycles of 95 °C for 15 s, 60 °C for 30 s, and (second step) 95 °C for 60 s; then 60 °C for 30 s. The reaction was then held at 25 °C. Each PCR was followed by obtaining melting curves to ensure single product amplification. As standard curves, serial dilutions of appropriate cDNA were used for gene quantification. The expression ratio of each gene to SUZ12 polycomb repressive complex 2 subunit (SUZ12) mRNA, which has been demonstrated to be suitable for normalization in bovine endometrial tissue [46], was calculated to adjust for any variations in the PCR reaction. Use of the Mx3000P real-time PCR analyzing system at elevated temperatures resulted in reliable and sensitive quantification of the RT-PCR products with high linearity (Pearson correlation coefficient *r* > 0.96). To exclude any contaminating genomic DNA, all experiments included controls that lacked the reverse transcription enzyme. As a negative control, water was used instead of RNA for the PCR to exclude any contamination from buffers and tubes.

### 4.4. Immunohistochemistry

Immunohistochemistry for CCR1, CCR2, CCR3, and CXCR3 in the bovine endometrium and fetal trophoblast on day 18 of pregnancy was performed using the automated Ventana HX System Discovery with a DabMapKit (Roche Diagnostics), as described previously [47]. The 5 µm-thick sections from paraffin-embedded tissue were incubated at room temperature with rabbit polyclonal anti-human CCR1 antibody (ab140756, Abcam PLC, Cambridge, UK; dilution 1:200), rabbit polyclonal anti-human CCR2 antibody (NBP1-48337, Novus Biologicals LLC, Littleton, CO, USA; dilution 1:100), rabbit polyclonal anti-human CCR3 antibody (251536, Abbiotec LLC, San Diego, CA, USA; dilution 1:50), or rabbit polyclonal anti-mouse CXCR3 antibody (orb5924, Biorbyt Ltd., Cambridge, UK; dilution 1:50) for 12 h. The signals were detected using anti-rabbit IgG-Biotin conjugate (Sigma-Aldrich Co., LLC, St. Louis, MO, USA) diluted 1:100 for 1 h, and then counterstained with hematoxylin. Negative controls were performed using normal rabbit IgG (NBP2-24891, Novus Biologicals) diluted at concentrations equivalent to the primary antibodies. The sections were observed with a Leica DMRE HC microscope (Leica Microsystems K.K., Tokyo, Japan) and photographed with a Nikon Digital Sight DS-Fi1-L2 (Nikon Instruments Co., Tokyo, Japan).

### 4.5. Endometrial Tissue Culture

For the tissue culture study, endometrial tissue samples were collected from cows on days 10–12 of the estrous cycle (*n* = 5 animals). The endometrial tissues from the bovine uterus were separated using a modification of procedures described previously [36,42]. The uterine lumen was washed three times with 30–50 mL of sterile physiological saline supplemented with 100 IU/mL penicillin, 100 µg/mL streptomycin, and 0.1% BSA (735078, Roche Diagnostics). The uterine horn was cut transversely with scissors into several segments, which were slit to expose the endometrial surface. Caruncular endometrial strips were dissected from the myometrial layer with a scalpel and washed once in 50 mL of sterile saline-containing antibiotics. The endometrial strips were then cut into small pieces (2 mm^3^). The tissues (approximately 20–30 mg wt) were pre-incubated in Dulbecco’s Modified Eagle’s medium (DMEM; D1152, Sigma-Aldrich Co.) supplemented with 0.1% BSA. After pre-incubating for 4 h, the endometrial tissues were placed in culture medium (DMEM/Ham’s F-12; 1:1 (*v*/*v*); D8900, Sigma-Aldrich Co.) supplemented with 10% (*v*/*v*) calf serum (C6278, Sigma-Aldrich Co.), 20 IU/mL penicillin, 20 µg/mL streptomycin, and 0.05 µg/mL amphotericin B (516104, EMD Millipore Corp. Billerica, MA, USA), and cultured at 37.5 °C in a humidified atmosphere of 5% CO_2_ in air. Cultured endometrial tissues were further incubated in the medium with recombinant proteins as follows: bovine CCL2 (RP0027B, Kingfisher Biotech., Inc. St. Paul, MN, USA), human CCL8 (281-CP, R&D Systems, Inc. Minneapolis, MN, USA), bovine CCL11 (RP0071B, Kingfisher Biotech.), human CCL14 (1578-HC, R&D Systems), human CCL16 (TP723266, OriGene Technologies, Inc., Rockville, MD, USA), bovine CXCL10 (RP0079B, Kingfisher Biotech.), bovine tumor necrosis factor-α (TNF; 2279-BT, R&D Systems), human leukemia inhibitory factor (LIF; TP723270, OriGene Technologies), bovine IFNT (1.1 × 10^5^ U/mg, generated from HEK293 cells as described previously; Takahashi et al., 2017 [48]) or supernatant derived from homogenized fetal trophoblast on day 18 of pregnancy (FMP). After incubation for 18 h, the endometrial tissue and supernatant were collected and stored at −80 °C until use.

To determine the responsibility of cultured endometrial tissue, the concentrations of PGE2 and PGF2α in the culture media after stimulation with TNF were examined using commercial ELISA kits (PGE2; 500141, PGF2α; 516011, Cayman Chemical Co., Ann Arbor, MI, USA). Both the PGE2 (299.7% ± 7.4%) and PGF2α (185.2% ± 16%) concentrations were significantly increased by the addition of TNF.

### 4.6. Statistical Analyses

Microarray data were analyzed statistically with an unpaired Student’s *t*-test and summarized using GeneSpring 12 (Agilent Technologies). The differences of mRNA expression in the endometrium between the non-pregnant and pregnant group (each day 15 and 18) were analyzed statistically with an unpaired Student’s *t*-test. In tissue culture experiments, the difference of mRNA expression in the endometrial tissues between control group and treated group was analyzed using one-way ANOVA with Dunnett’s Multiple Comparison post hoc test with the KaleidaGraph 3.6 (Synergy Software, Reading, PA, USA) software package. All Experimental data for real-time PCR are presented as the mean ± SEM. A *p*-value < 0.05 was considered statistically significant.

## 5. Conclusions

**Figure 6:**
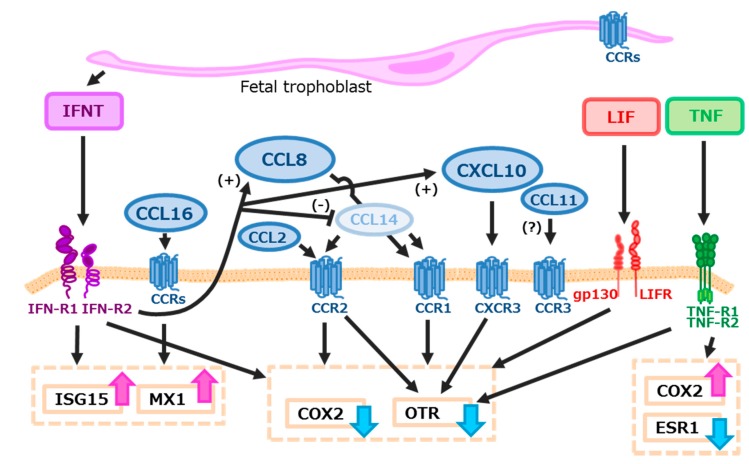
Hypothetical model for inhibition of luteolysis by IFNT and chemokines. Although this model is not concerned with the effects of steroids or growth factors, IFNT, CCL2, CCL8, CCL16, CXCL10, and LIF may block TNF-stimulated-COX2 expression in bovine endometrial cells, leading to the reduction of TNF-induced PGF2α output from the cells. Furthermore, IFNT and CCL16 may stimulate anti-viral activity by up-regulating ISG15 and MX1 expression at the time of maternal recognition in cows. Red and blue arrows show stimulatory and inhibitory actions of each substance, respectively. IFNT may stimulate both CCL8 and CXCL10 production and inhibit CCL14 production from bovine endometrium. Effects of CCL11 on bovine endometrial function are still unclear, although its receptor (CCR3) is expressed in the endometrial epithelial cells.

In conclusion, chemokines that are increased in the endometrium of early pregnant cows, including CCL2, CCL8, CCL11, CCL14, CCL16, and CXCL10, may regulate PG production and antiviral activity in the uterus at the time of maternal recognition, together with IFNT, TNF, or LIF (Figure 6).

## Figures and Tables

**Table 1:** Comparison of mRNA levels for selected chemokines in the endometrium of pregnant vs. non-pregnant cows as determined by microarray analysis (Fold > 2.0; *p* < 0.05).

Genes	Day 15 (344 Genes)	Day 18 (1336 Genes)
*CCL2* (*MCP-1*)	3.58	-
*CCL8* (*MCP-2*)	4.91	12.9
*CCL11* (*Eotaxin*)	-	61.1
*CCL14* (*HCC-1*)	2.67	-
*CCL16* (*HCC-4*)	2.55	-
*CXCL10* (*IP-10*)	2.10	21.1

**Table 2:** Primers used in real-time PCR.

Genes	Sequence (5′-3′)	GenBank Accession Number	Size (bp)
*CCL2*	Forward: TGCAGACCCCAAGCAGAAAT	NM_174006	144
	Reverse: AGAGGGCAGTTAGGGAAAGC		
*CCL8*	Forward: AACATGAAGGTCTCCGCTGG	NM_174007	108
	Reverse: GCAGCAGGTGATTGGGGTAG		
*CCL11*	Forward: TCACGAGCAGCAAATGTCCT	NM_205773	101
	Reverse: CATGGCATTCTGGACCCACT		
*CCL14*	Forward: ACTAAATTTCCCCGCTCGCT	NM_001046585	121
	Reverse: TGGCCAAACTTCTGCAGAGT		
*CCL16*	Forward: GCCCACTGAGAGGATGAAGG	XM_002695627	129
	Reverse: TACTTCAGGCAGCAGTTGGG		
*CXCL10*	Forward: CTCGAACACGGAAAGAGGCA	NM_001046551	117
	Reverse: TCCACGGACAATTAGGGCTT		
*ISG15*	Forward: GCAGACCAGTTCTGGCTGTCT	NM_174366	58
	Reverse: CCAGCGGGTGCTCATCAT		
*MX1*	Forward: GAGGTGGACCCCCAAGGA	NM_173940	58
	Reverse: CCACCAGATCGGGCTTTGT		
*COX2*	Forward: TGTGAAAGGGAGGAAAGAGC	AF004944	115
	Reverse: GGCAAAGAATGCAAACATCA		
*OTR*	Forward: TGTGCTGGACGCCATTCTT	NM_174134	93
	Reverse: GGAGCATGGCGATGATGAAAG		
*ESR1*	Forward: CAGGCACATGAGCAACAAAG	NM_001001443	84
	Reverse: TCCAGCAGCAGGTCGTAGAG		
*SUZ12*	Forward: GAACACCTATCACACACATTCTTGT	NM_001205587	130
	Reverse: TAGAGGCGGTTGTGTCCACT		

## Abbreviations

IFNT	interferon τ
COX2	cyclooxygenase 2
OTR	oxytocin receptor
ESR1	estrogen receptor α
CL	corpus luteum
PG	prostaglandin
TNF	tumor necrosis factor α
LIF	leukemia inhibitory factor
FMP	supernatant derived from homogenized fetal trophoblast
MCP	monocyte chemoattractant protein
MIP	macrophage inflammatory protein
E2	estradiol-17β
